# Crystal Structures of the Network-Forming Short-Arm Tips of the Laminin β1 and γ1 Chains

**DOI:** 10.1371/journal.pone.0042473

**Published:** 2012-07-31

**Authors:** Federico Carafoli, Sadaf-Ahmahni Hussain, Erhard Hohenester

**Affiliations:** Department of Life Sciences, Imperial College London, London, United Kingdom; Universität Erlangen-Nürnberg, Germany

## Abstract

The heterotrimeric laminins are a defining component of basement membranes and essential for tissue formation and function in all animals. The three short arms of the cross-shaped laminin molecule are composed of one chain each and their tips mediate the formation of a polymeric network. The structural basis for laminin polymerisation is unknown. We have determined crystal structures of the short-arm tips of the mouse laminin β1 and γ1 chains, which are grossly similar to the previously determined structure of the corresponding α5 chain region. The short-arm tips consist of a laminin N-terminal (LN) domain that is attached like the head of a flower to a rod-like stem formed by tandem laminin-type epidermal growth factor-like (LE) domains. The LN domain is a β-sandwich with elaborate loop regions that differ between chains. The γ1 LN domain uniquely contains a calcium binding site. The LE domains have little regular structure and are stabilised by cysteines that are disulphide-linked 1–3, 2–4, 5–6 and 7–8 in all chains. The LN surface is not conserved across the α, β and γ chains, but within each chain subfamily there is a striking concentration of conserved residues on one face of the β-sandwich, while the opposite face invariably is shielded by glycans. We propose that the extensive conserved patches on the β and γ LN domains mediate the binding of these two chains to each other, and that the α chain LN domain subsequently binds to the composite β-γ surface. Mutations in the laminin β2 LN domain causing Pierson syndrome are likely to impair the folding of the β2 chain or its ability to form network interactions.

## Introduction

**Figure 1:**
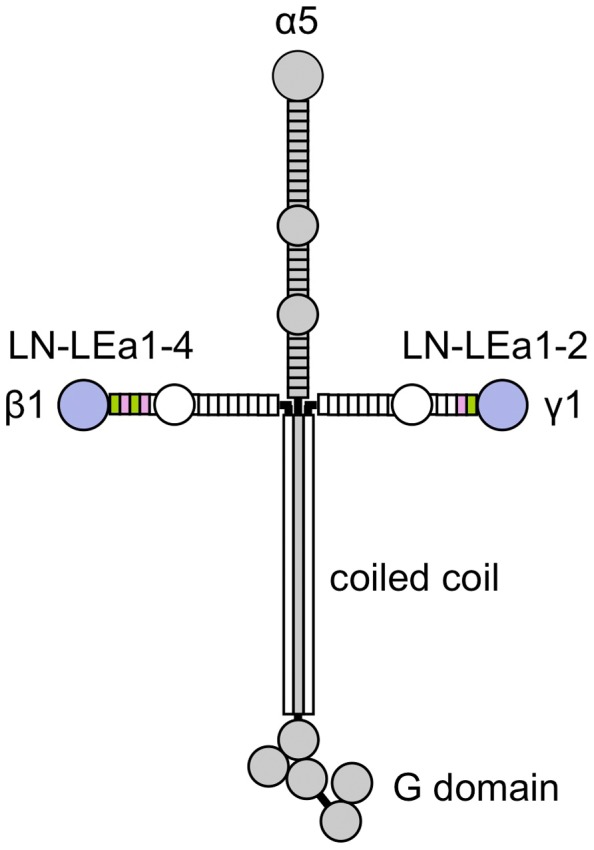
Schematic drawing of the laminin-511 heterotrimer. The α5 chain is shown in grey. In the β1 and γ1 chain, the regions corresponding to the crystal structures described in this report are coloured. LN, laminin N-terminal domain; LE, laminin-type epidermal growth factor-like domain.

Basement membranes are sheet-like extracellular matrices that underlie all epithelia and endothelia, and surround muscle, peripheral nerve and fat cells. They are found in all metazoa and have many critical functions in tissue development and homeostasis [1], [2]. The major constituents of basement membranes are laminin, collagen IV, nidogen, perlecan and collagen XV/XVIII; this invariant set of glycoproteins has been called the “basement membrane toolkit” [3]. The laminins constitute a family of heterotrimeric glycoproteins. The five α, three β and three γ chains encoded by the human genome give rise to at least 16 laminin isoforms [4], [5], which play numerous important roles in embryo development, organ function and human disease [6]. The archetypal laminin-111 (α1β1γ1) is a cross-shaped molecule [7]. The long arm of the cross is a coiled coil of all three chains, terminating in the cell-adhesive G domain. The short arms consist of one chain each and are composed of a distal laminin N-terminal (LN) domain followed by long tandem repeats of laminin-type epidermal growth factor-like (LE) domains, interrupted by one or two globular domains of unknown function (Fig. 1). The LN domains are crucial for laminin polymerisation into a cell-associated two-dimensional network [8]–[11]. Eight of the 16 known laminin heterotrimers (111, 121, 211, 221, 213, 511, 521 and 523) have a full complement of LN domains [4] and are predicted to form polymers, but this has been shown experimentally only for a few representatives [12]. Mutations in the LN domains of the laminin α2 and β2 chain cause, respectively, muscular dystrophy and glomerular kidney disease, highlighting the importance of the short-arm tips for laminin function [13]–[15].

The structural basis of laminin network formation is largely unknown. A seminal study with proteolytic fragments of laminin-111 established that laminin polymerisation requires calcium ions and that all three arms contribute to the nodes in the network (“three-arm interaction model”) [11]. This mechanism is supported by more recent experiments using recombinant laminin heterotrimers with deleted or swapped LN domains [10]. However, the three-arm interaction model was challenged by a study using recombinant LN-LEa1–4 fragments, which showed a promiscuous binding repertoire, including α chain self-interactions [16]. We recently reported the first crystal structure of a laminin short-arm tip, namely that of the α5 LN-LEa1–2 fragment [17]. This structure confirmed the predicted β-sandwich fold of the LN domain [18] and showed that the LN domain interacts tightly with the first LE domain. A patch on one face of the LN domain was found to be conserved in all laminin α chains, and mutations in this patch abolished the ability of the crystallised protein to inhibit laminin-111 polymerisation *in vitro* [17]. This result suggested that the conserved patch is involved in laminin network formation and prompted us to carry out surface plasmon resonance experiments similar to those of Odenthal et al. [16]. We did not observe the reported α5-β1 and α5-γ1 interactions, but detected a weak interaction between the β1 and γ1 short-arm tips. Addition of the α5 short-arm tip to the β1-γ1 complex resulted in the formation of a stable ternary complex [17], in full agreement with the three-arm interaction model [11]. We have recently confirmed these results in solution and with other laminin α chains (manuscript in preparation).

Even though important insights could be gained from the laminin α5 LN-LEa1–2 structure, it remained important to determine representative structures of the short-arm tips of the laminin β and γ chains. First, because of the considerable divergence of laminin chains, the α5 LN-LEa1–2 structure is a relatively poor template for the corresponding regions of the β and γ chains (Table 1). Second, a segment of 50 residues, corresponding to a functionally critical loop in the related netrins G1 and G2 [19], was disordered in the laminin α5 LN-LEa1–2 structure. Third, a recent mass spectrometric analysis [20] suggested that the disulphide linkage pattern in the LEa domains of the laminin β1 chain might differ from the [1–3, 2–4, 5–6, 7–8] pattern established by the crystal structure of laminin γ1 LEb2–4. Here, we report crystal structures of the N-terminal regions of the laminin β1 and γ1 chains, which define the common and unique features of laminin short-arm tips and reveal likely interaction sites for laminin polymerisation.

**Table 1:** Pairwise sequence identities between LN-LEa1-2 regions of mouse laminin chains.

	α1	α2	α3B	α5	β1	β2	γ1	γ3
**α1**	—	72.0	49.9	49.6	30.3	28.8	30.1	33.3
**α2**		—	48.1	50.3	31.0	29.4	30.6	33.3
**α3B**			—	67.7	28.6	27.5	30.7	33.1
**α5**				—	28.4	30.8	32.3	34.3
**β1**					—	70.4	30.2	30.6
**β2**						—	28.9	30.8
**γ1**							—	63.7
**γ3**								—

## Results

### Crystal structure of laminin β1 LN-LEa1–4

We obtained crystals of an untagged laminin β1 chain construct spanning the LN domain, LEa domains 1–4 and the first half of the fifth LE domain (domains VI and V in the old laminin nomenclature [4]). The crystals diffracted isotropically to 3.1 Å resolution and the structure was determined by molecular replacement and refined to a free R-factor of 0.288 (Table 2). The partial LEa domain at the C-terminus is disordered and the structure is therefore referred to as β1 LN-LEa1–4 in this report.

**Table 2:** Crystallographic statistics.

Data set	Laminin β1 LN-LEa1–4	Laminin γ1 LN-LEa1–2
*Data collection statistics*		
Space group	C222_1_	R32
Unit cell dimensions		
a, b, c (Å)	143.46, 152.50, 92.70	203.01, 203.01, 93.69
α, β, γ (°)	90, 90, 90	90, 90, 120
Solvent content (%)	72	72
Resolution (Å)	50-3.10 (3.27-3.10)^a^	50-3.17 (3.25-3.17)
R_merge_	0.089 (0.664)	0.098 (0.674)
<I/σ(I)>	12.9 (2.7)	18.6 (4.5)
Completeness (%)	99.5 (99.3)	99.4 (100)
Multiplicity	6.0 (6.1)	11.0 (11.2)
*Refinement statistics*		
Resolution (Å)	20-3.1	20-3.2
Reflections	18789	12215
Atoms	3578	2742
R_work_/R_free_	0.247/0.288	0.249/0.297
R.m.s. deviation bonds (Å)	0.009	0.008
R.m.s. deviation angles (°)	1.5	1.5
R.m.s. deviation B-factors (Å^2^)^b^	1.7	1.5
Average B-factor (Å^2^)	90.8	93.7
Ramachandran plot (%)^c^	70.6, 27.8, 1.5, 0.0	68.0, 28.2, 2.6, 1.3

a Values in parantheses are for the highest resolution shell.

b Difference in B-factors of atoms connected by a covalent bond.

c Residues in core, allowed, generously allowed and disallowed regions of the Ramachandran plot [44].

**Figure 2:**
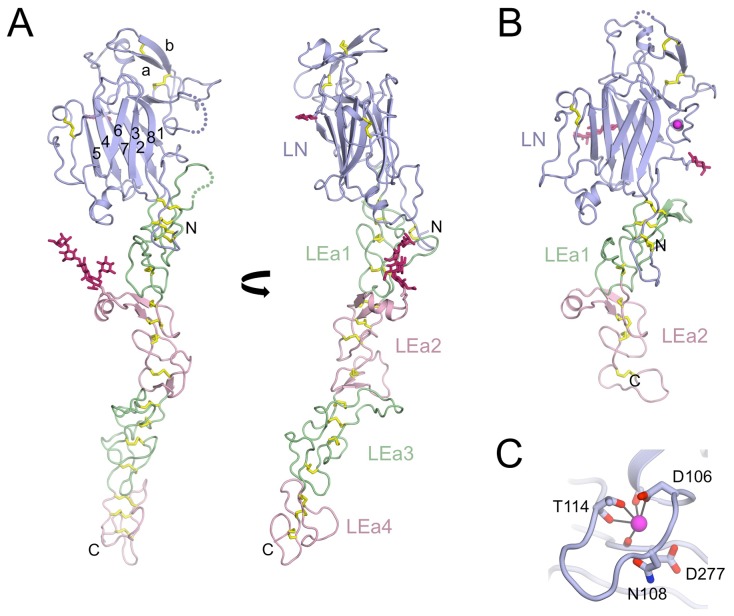
Crystal structures of the short-arm tips of the laminin β1 and γ1 chains. (A) Two orthogonal views of the laminin β1 LN-LEa1–4 structure. The LN domain is in blue, and the LEa domains are in green (LEa1 and LEa3) and pink (LEa2 and LEa4). Disulphide bridges and *N*-linked glycans are in yellow and dark pink, respectively. Loop regions without electron density are indicated by dotted lines. The β-strands in the LN domain are labelled. (B) Laminin γ1 LN-LEa1–2 structure. The colours used are the same as in (A). A calcium ion is shown as a magenta sphere. (C) Details of the calcium binding site in laminin γ1 LN-LEa1–2. The calcium ion is co-ordinated by the side chains of Asp106 and Thr114, as well as the carbonyl groups of residues 103, 114 and 276.

**Figure 3:**
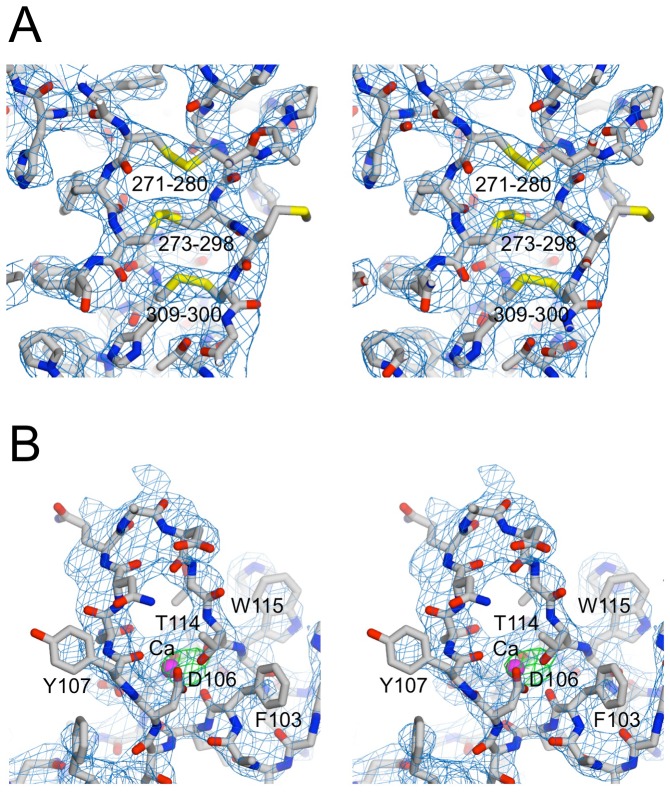
Simulated annealing omit maps of selected model regions. Partial models, from which regions of interest were omitted, were subjected to simulated annealing refinement from 3000 K to remove model bias. Shown are SIGMAA-weighted 2F_obs_-F_calc_ maps calculated from the partial models after refinement. (A) Stereoview of LEa1 in the laminin β1 LN-LEa1–4 structure. Residues 270–310 were omitted from the map calculation (R_free_ = 0.333). The disulphide bridges are labelled with the sequence numbers of the linked cysteines. According to the mass spectrometric analysis of Kalkhof et al. [20], Cys271 is linked to Cys273, Cys280 unpaired, Cys298 linked to Cys300, and Cys309 linked to Cys313 (not visible). (B) Stereoview of the calcium binding loop in the laminin γ1 LN-LEa1–2 structure. Residues 102–115 and the calcium ion were omitted from refinement (R_free_ = 0.315). The green electron density is a F_obs_-F_calc_ difference density map contoured at 7σ, confirming the presence of a heavy atom at the position of the calcium ion.

The most striking feature of the laminin β1 LN-LEa1–4 structure is the straight ∼100 Å-long stalk made up of the four LE domains (Fig. 2A). Apart from one short α-helix and four short β-strands, the LEa1–4 tandem consists only of irregular loops. Lacking a conventional hydrophobic core, the LEa1–4 tandem is stabilised by a near-continuous ladder of 16 disulphide bridges. Within each LE domain, the eight cysteines are linked in the same [1–3, 2–4, 5–6, 7–8] pattern as in the crystal structures of laminin γ1 LEb2–4 [21] and laminin α5 LN-LE1-2 [17] (Fig. 3A). Thus, our structure does not confirm the alternative pattern determined by mass spectrometric analysis of laminin β1 LN-LEa1–4 [20].

The LN domain is attached to the LEa1-4 tandem like the head of a sunflower, with a pronounced ∼35° kink at the junction (Fig. 2A). The total length of the β1 LN-LEa1–4 structure is ∼150 Å and the LN head measures ∼55 Å at its widest point. As already described for the laminin α5 chain [17] and the related netrins G1 and G2 [19], [22], the LN domain is a β-sandwich of jelly roll topology [23]. The eight strands of the jelly roll are arranged in two sheets, β1-β2-β7-β4-β5 and β6-β3-β8. This basic structure is elaborated by several insertions that add a number of α-helices and a long β-hairpin containing two disulphide bridges, which lies across the top edge of the LN domain (in the view of Fig. 2A). The N-terminus of the β1 LN domain, which starts with a disulphide-bonded reverse turn, interacts tightly with the LEa1 domain. A similar interaction of the N-terminal segment was seen in the laminin α5 LN-LEa1–2 and netrin G structures [17], [19], [22] and most likely explains why LN domains do not fold in isolation [16], [24]. There are two *N*-linked glycosylation sites in the laminin β1 LN-LEa1–4 structure, one at Asn120 at the N-terminus of the β2 strand of the LN domain, and another at Asn356 in the α-helical protrusion of LEa2. The shielding of the β1-β2-β7-β4-β5 face by a glycan appears to be a common feature of all LN domains [17], [19], [22].

### Crystal structure of laminin γ1 LN-LEa1–2

We also obtained crystals of a His-tagged laminin γ1 construct spanning the LN-LEa1–2 region. The diffraction limit of the trigonal crystals was limited to ∼3.8 Å in the c* direction, but extended beyond 3.2 Å in the directions normal to c*. Despite the modest resolution of the diffraction data along c*, a complete laminin γ1 LN-LEa1–2 model could be built and refined to a free R-factor of 0.297 (Table 2). The overall structure of laminin γ1 LN-LEa1–2 is similar to that of the corresponding region in the laminin β1 chain (Fig. 2B), albeit with differently distributed glycosylation sites (the modified residues in the γ1 LN domain are Asn58 just before the β1 strand, and Asn132 in the β2 strand). A significant difference between the laminin β1 and γ1 structures is the presence of a calcium ion in the γ1 LN domain (Figs. 2C and 3B). The calcium ion is bound at the C-terminus of a short α-helix and co-ordinated by the side chains of Asp106 and Thr114, as well as three main chain carbonyl oxygens assume that the co-ordination shell is completed by water molecules bridging to the side chains of Asn108 and/or Asp277. An equivalent calcium site is present in netrins G1 and G2 [19], [22], but in these proteins there is a direct bond to the metal ion from a glutamic acid replacing Asn108 of laminin β1.

### Comparison of the LN-LEa1–2 regions of the laminin α5, β1 and γ1 chains

**Figure 4:**
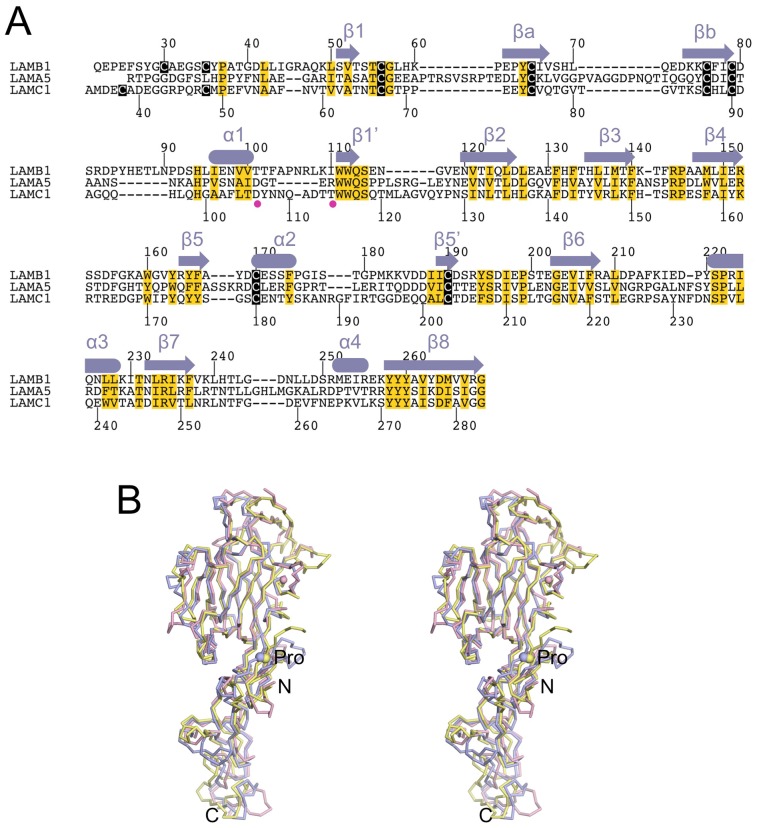
Comparison of the short-arm tips of the laminin α5, β1 and γ1 chains. (A) Structure-based sequence alignment of the LN domains of the mouse laminin α5, β1 and γ1 chains. The sequence numbering of the β1 and γ1 chains is indicated above and below the alignment, respectively. Cysteines are in white on black background and structurally important residues are shaded yellow. Residues co-ordinating the calcium ion the γ1 LN-LEa1–2 structure are marked by magenta filled circles. The secondary structure elements common to all three structures are shown above the alignment. (B) Stereoview of a superposition of the crystal structures of the LN-LEa1–2 regions of the laminin α5 chain [17] (blue), the laminin β1 chain (yellow) and the laminin γ1 chain (pink). The structures were superimposed on the central β-strands of the LN domain. The view direction is the same as in Figure 2B. The conserved proline in the N-terminal segment (see text) is indicated by a sphere and labelled. The N- and C-terminus is labelled as well.

A structure-based sequence alignment of the LN domains of the laminin α5, β1 and γ1 chains is shown in Figure 4A. This alignment differs from that previously published [17] in the region of the calcium binding site and near the β5 strand. The secondary structure elements of the LN domain are well conserved across all laminin chains, but there are differences in the connecting loop regions, in particular in the regions shaping the top of the LN domain (βa-βb hairpin, β7-α4 loop). The previously unassigned region of the laminin α5 chain maps to the βa-βb hairpin, which has unusually long connections in the α5 chain (Fig. 4A).

A superposition of the three laminin short-arm structures is shown in Figure 4B. The β-sandwiches of the three LN domains are very similar, and the few substantial differences are confined to peripheral regions. Pairwise superpositions of the LN domains with SSM [25] gave r.m.s. deviations of 1.72 Å for the α5-β1 pair (185 Cα atoms), 1.50 Å for the α5-γ1 pair (192 Cα atoms), and 1.67 Å for the β1-γ1 pair (215 Cα atoms). The three structures are also strikingly similar in the relative orientations of their LN and LEa1 domains (Fig. 4B). This is remarkable, given that the interactions between the LN domains and the protruding loops of the LEa1 domain are not conserved (not shown). It seems that the conformation of the LN-LEa1 junction is fixed by the extended N-terminal segment of the LN domain, which inserts a strictly conserved proline into a pocket created by the 1–3 and 2–4 disulphide bridges of the LEa1 domain [17] (Fig. 4B).

### Location of Pierson syndrome mutations

**Figure 5:**
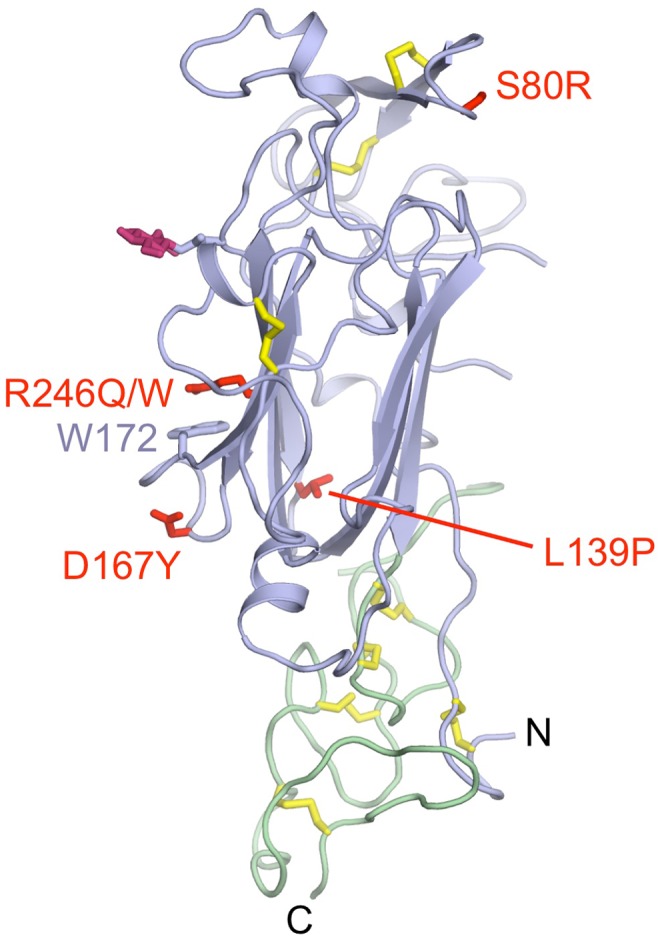
Location of Pierson syndrome mutations. Shown is a cartoon drawing of the first two domains of the laminin β1 LN-LEa1-4 structure (LN, blue; LEa1, green; disulphide bridges, yellow; *N*-linked glycan; dark pink), in which residues corresponding to those mutated in the laminin β2 chain of Pierson syndrome patients [15] are shown in red. Only homozygous missense mutations are shown. The view direction is similar to the right panel of Figure 2A.

Mutations in the human *LAMB2* gene cause Pierson syndrome, a severe glomerular kidney disease accompanied by ocular and neurological abnormalities [26], [27]. Similar pathological features are observed in mice lacking the laminin β2 chain [28], [29], whose renal defects can be rescued by overexpression of the laminin β1 chain [30]. The majority of mutations causing Pierson syndrome result in premature stop codons; these mutations are distributed over the entire laminin β2 chain. In contrast, the few pathogenic missense mutations are concentrated in the LN domain [15]. Figure 5 shows the missense mutations mapped onto the structure of the mouse β1 LN domain, which is 72% identical to the human β2 LN domain.

R246W is a recurrent mutation with a severe phenotype [15], [26] and a dramatic reduction in laminin β2 protein [26]. Similarly, the rarer R246Q mutation has been shown to impair laminin β2 secretion in a mouse model of Pierson syndrome [31]. Arg246 is located on the β7 strand of the β2 LN domain, flanked on one side by Trp172 and on the other side by a glycosylation site at Asn248. The arginine and tryptophan are conserved in the β1 LN domain, but the glycosylation site in β1 LN is on the adjacent β2 strand at Asn120 (Fig. 5). Given the invariant presence of a glycosylation site on this face of the LN domain (see above), it is plausible that the R246W and R246Q mutations affect the folding and/or secretion of the laminin β2 chain. The remaining homozygous missense mutations in Pierson syndrome are S80R, L139P and D167Y [15]. They affect, respectively, a conserved serine in the βa-βb hairpin at the top of the LN domain (Ser 68 in β1 LN), a conserved leucine in the hydrophobic core of the LN domain (Leu127 in β1 LN), and a conserved aspartic acid stabilising the loop connecting strands β4 and β5 (Asp155 in β1 LN) (Fig. 5). The L139P mutation is likely to have a dramatic effect on folding, whereas the other two mutations are less obviously disruptive. The D167Y mutation is adjacent to Trp172, which in turn interacts with Arg246 (Fig. 5). Thus, this region of the LN domain may be particularly sensitive to mutational disruption. Finally, there are two further missense mutations that so far have only been observed in compound heterozygotes: S179F and C321R [15]. The former mutation maps to the β5 strand (Ala167 in β1 LN) and the latter disrupts a disulphide bridge in LEa1.

### Surface conservation in the short-arm tips of the laminin α5, β1 and γ1 chains

Laminins are members of the basement membrane toolkit present in all metazoa [3]. The minimal functional set of laminin chains appears to consist of two α, one β and one γ chain, which can assemble into two network-forming laminins. If one assumes that the mechanism of laminin polymerisation has been conserved during evolution, mapping the degree of sequence conservation onto our structures of laminin short-arm tips might reveal the functionally important surface regions.

**Figure 6:**
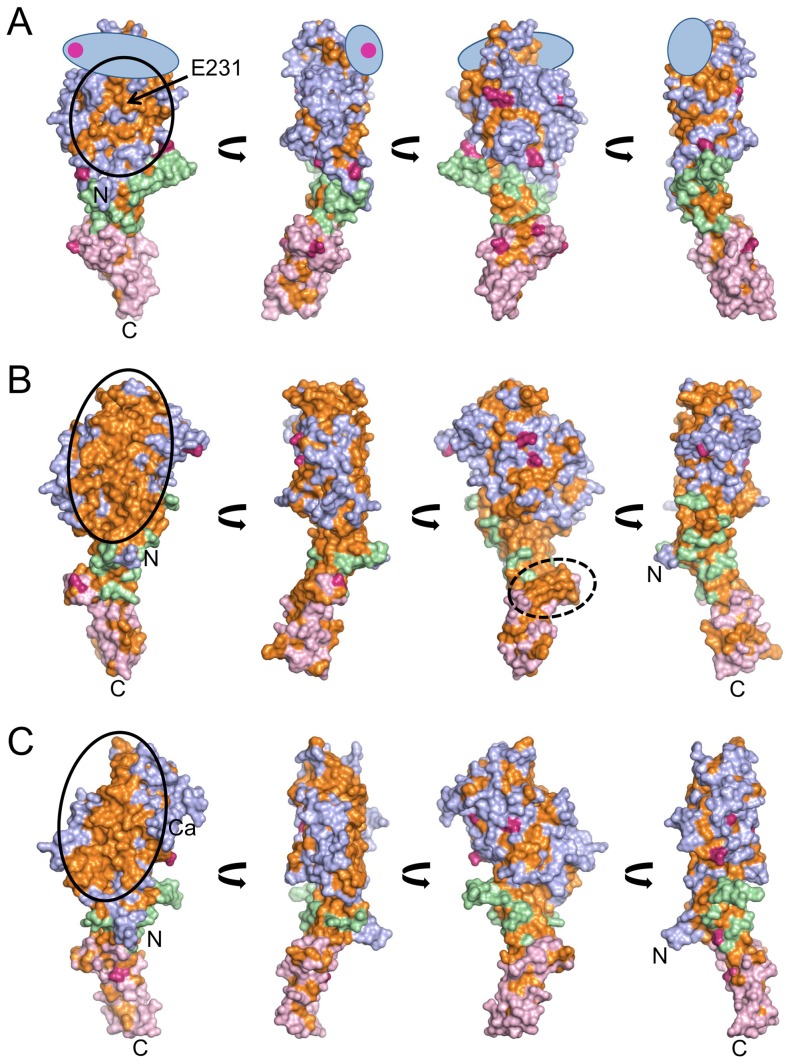
Conserved surface regions in the short-arm tips of the laminin α5, β1 and γ1 chains. Shown are surface representations of the LN-LEa1–2 regions of (A) the laminin α5 chain [17], (B) the laminin β1 chain, and (C) the laminin γ1 chain. The four views of each chain are related by 90° rotations about the vertical axis, with the leftmost view being similar to that of Figure 2B. The LN, LEa1 and LEa2 domains are in blue, green and pink. Strictly conserved residues are shown in orange. Residues that are predicted to be modified by *N*-linked glycosylation in at least one homologue are shown in dark pink. In (A) the approximate location of the region missing in the laminin α5 LN-LEa1–2 structure is indicated by a blue oval, and the arrow points to the functionally important Glu231 residue [17]. The conserved patch on the β3-β6-β8 sheet is marked by an oval solid line. The conserved ridge in the LEa2 domain of the laminin β1 chain (see text) is marked by an oval broken line. The positions of the N- and C-termini and the calcium ion are indicated. For the laminin β and γ subfamilies (two members each), three species were included in the analysis. For the more divergent α subfamily (four members), only two species were included in the analysis. The following sequences were used: mouse α1 (NCBI reference NP_032506), mouse α2 (NP_032507), mouse α3B (NP_034810), mouse α5 (NP_001074640), chicken α1 (NP_001186735), chicken α2 (XP_419746), chicken α3 (XP_426078), chicken α5 (XP_003642506, completed using UniProt entry F1NZZ2), mouse β1 (NP_032508), mouse β2 (NP_032509), chicken β1 (XP_415943), chicken β2 (NP_989497), zebrafish β1 (NP_775382), zebrafish β2 (NP_001229974), mouse γ1 (NP_034813), mouse γ3 (NP_035966), chicken γ1 (XP_001234659), chicken γ3 (XP_415462), zebrafish γ1 (NP_775384), zebrafish γ3 (XP_687343).

The few residues that are identical in all laminin chains (14% in the LN-LEa1–2 region) appear to be conserved for structural reasons (not shown). However, sequence comparisons within the α, β and γ chain subfamilies reveal an intriguing conservation of residues on the surface-exposed face of the β6-β3-β8 sheet of the LN domain (Fig. 6). Equally striking is that the opposite face of the LN domain (β1-β2-β7-β4-β5 sheet) carries at least one *N*-linked glycan in every laminin chain (Fig. 6). These observations suggest that the β6-β3-β8 surface mediates critical interactions in all three chains. We previously confirmed this hypothesis for the α5 chain by site-directed mutagenesis of Leu230, Glu231 and Glu234 [17]. Our new results show that the disordered βa-βb hairpin in the laminin α5 LN-LEa1-2 structure is close to, but unlikely to obstruct, the conserved patch in laminin α chains (Fig. 6A). The conserved patch is most extensive in the laminin β1 and β2 chains, extending to and including most of the top surface of the LN domain (Fig. 6B). The conserved patch in the γ1 and γ3 chains includes less of the top surface of the LN domain, but is otherwise very similar to that of the β chain subfamily (Fig. 6C). Importantly, very few surface residues are conserved across the β and the γ chain subfamilies (not shown). Thus, the same general surface region of the LN domain is conserved in each of the three subfamilies, but the conserved residues are unique to each subfamily. The level of surface conservation is generally lower in the LE domains, with the exception of a pronounced patch on a protruding ridge in the LEa2 domain of the β chains (third view of Fig. 6B). This ridge corresponds to the first β-hairpin and helical loop of LE2a (laminin β1 residues 345–350, 353, and 361–363).

## Discussion

A large body of biochemical and genetic data implicate the N-terminal short-arm tips of laminin heterotrimers in network formation and basement membrane assembly. The present results, combined with our previous study [17], provide a complete structural description of these critical laminin regions. Despite pairwise sequence identities of only ∼30%, the N-terminal regions of the laminin α5, β1 and γ1 chains are structurally very similar. The LN domain is attached to the LEa tandem like the head of a flower to the stem, with a pronounced angle at the junction. This arrangement appears to be fairly rigid, given that very similar angles were observed in crystal structures of the α5, β1 and γ1 short-arm tips. The laminin β1 LN-LEa1–4 structure affords the most complete view of any LE tandem to date and shows that the four LE domains form a linear, rod-like, array. The linear arrangement is the result of a conserved set of interactions at the LE interfaces, which was first identified in the laminin γ1 LEb2–4 structure [21]. The overall shape of the laminin β1 LN-LEa1–4 crystal structure agrees well with a low-resolution solution structure of the complete γ1 chain short arm [32].

The crystal structures also suggest new, testable, hypotheses about the mechanism of laminin polymerisation. As described in the Introduction, the available evidence favours a mechanism in which one α chain, one β chain and one γ chain associate to form the nodes in the laminin network. Whether the interactions in this ternary node are made exclusively by the LN domains or also involve the LEa domains is unclear and difficult to study, given that LN domains cannot be produced in isolation [16], [24]. Our previous surface plasmon resonance experiments with the α5, β1 and γ1 short arms suggested a hierarchy of network assembly, with the β1 and γ1 short arms forming a weak binary complex first, which is then strengthened by the slow incorporation of the α5 chain [17]. Interestingly, our present analysis of surface conservation in the laminin α, β and γ subfamilies shows that the extent of sequence conservation is much higher in the β and γ LN domains than in the α LN domains (Fig. 6), which may correlate with the higher stability of the β-γ binary complex compared with all other pairings [17]. We propose that the extensive conserved patches on the β and γ LN domains mediate the binding of these two chains to each other. We think that the α chain then binds to a composite β-γ surface using its own conserved patch, which our previous mutagenesis has shown to be important for laminin network formation [17]. Exactly how the three short arms interact in a network node is impossible to predict, of course. For steric reasons, it is difficult to imagine that a ternary contact can be formed by three flat surfaces, which suggests that at least one chain might contribute a region other than the conserved β6-β3-β8 sheet. The most likely candidate, in our opinion, is the β1 chain, which has a highly conserved region at the top of the LN domain in addition to the conserved patch on the β6-β3-β8 sheet (Fig. 6B). The top of the β1 LN domain is constructed from the βa-βb hairpin and the β7-α4 loop (Fig. 2A). The Pierson syndrome mutation S80R maps to the βa-βb hairpin (Fig. 5) and may impair the ability of the laminin β2 chain to form network interactions. It is worth noting that netrins G1 and G2, which are functionally unrelated to laminins, use the corresponding top region of their LN domains to bind to the leucine-rich repeats of NGLs [19].

The polymerisation of laminin-111 requires calcium [33], [34] and the same presumably applies to all other network-forming laminins. Equilibrium dialysis and dot blot experiments with proteolytic laminin-111 fragments suggested the presence of a calcium binding site in the γ1 chain, and little calcium binding to the α1 and β1 chains [11], [33]. This result is entirely consistent with our crystal structures, which show a calcium site only in the γ1 domain. Furthermore, the partial co-ordination shell of this calcium ion (Fig. 2C) is consistent with the relatively high dissociation constant of 55 µM determined by equilibrium dialysis [33]. A caveat is that the α5 and β1 short-arm tips were crystallised at low pH, and with no calcium added in the case of the β1 construct. However, sequence analysis of the putative calcium-binding loops suggests that the α5 and β1 chains cannot bind calcium in the same manner as the γ1 chain (Fig. 4A). Whether the calcium ion bound to the γ1 LN domain is responsible for the calcium-dependence of laminin polymerisation remains to be seen. The calcium-binding loop in the γ1 LN domain does not contribute to the conserved patch on the β6-β3-β8 sheet, but it is adjacent to it (Fig. 6C). It is also possible that the critical calcium binding sites are only formed in the ternary α1-β1-γ1 complex. Circular dichroism spectra of recombinant LN-LEa1–4 fragments in the absence or presence of 2 mM calcium show calcium-dependent changes in all three chains of laminin-111 [16], which may be due to calcium binding weakly to partial sites in the isolated chains.

In conclusion, we now have in hand representative structures of the short-arm tips of all three classes of laminin chains, which will provide a solid foundation for further investigations into the molecular mechanism of laminin polymerisation.

## Materials and Methods

### Expression vectors

We used pCEP-Pu expression vectors, which leave a vector-derived APLA sequence at the N-terminus of the encoded proteins [35]. The vector for the laminin β1 LN-LEa1–4 fragment codes for residues 21–542 of the mouse laminin β1 chain (UniProt P02469) [36]. The vector for the laminin γ1 LN-LEa1-2 fragment codes for residues 34–395 of the mouse laminin γ1 chain (UniProt P02468) and a C-terminal AAAHHHHHH sequence [17].

### Protein production

We used human embryonic kidney HEK293 c18 cells (ATCC) for protein production. The cells were grown at 37°C with 5% CO_2_ in Dulbecco's modified Eagle's medium/F12 (Invitrogen) containing 10% fetal bovine serum, 2 mM glutamine, 10 U/ml penicillin, 100 µg/ml streptomycin and 250 µg/ml geneticin. The cells were transfected with the expression vectors using Fugene (Roche Diagnostics) and selected with 1 µg/ml puromycin (Sigma). Confluent cells in a HYPERFlask (Corning) were washed twice with PBS and incubated with serum-free medium for 3 weeks, with weekly medium exchanges. The pooled serum-free conditioned medium containing the laminin β1 LN-LEa1–4 fragment was concentrated using a Vivaflow 200 cross-flow ultrafiltration unit (Sartorius) with a 30 kDa cut-off and dialysed against 50 mM MES pH 6.5, 100 mM NaCl. The dialysed sample was loaded onto a 6-ml RESOURCE Q column (GE Healthcare) using an Äkta Purifier (GE Healthcare). The protein was eluted with a step gradient (50 mM MES pH 6.5, 100 mM to 1 M NaCl), concentrated using a Vivaspin centrifugal device (Sartorius), and further purified on a Superdex 200 HR10/30 size exclusion chromatography column (GE Healthcare) using 5 mM MOPS pH 6.8, 200 mM NaCl as the running buffer. The final yield was 4 mg of β1 LN-LEa1–4 protein from 1.5 litres of cell culture medium. The pooled serum-free conditioned medium containing the laminin γ1 LN-LEa1–2 fragment was loaded onto a 5-ml HisTrap column (GE Healthcare). The protein was eluted with 500 mM imidazole in HBS-Ca buffer (20 mM Na-HEPES pH 7.5, 150 mM NaCl, 2 mM CaCl_2_), concentrated, and further purified on a Superdex 200 HR10/30 column using HBS-Ca as the running buffer. The final yield was 2 mg of γ1 LN-LEa1–2 protein from 1 litre of cell culture medium.

### Crystallisation

Crystal screening was done with a Mosquito nanolitre robot (TTP LabTech). Initial crystals of the laminin β1 LN-LEa1–4 fragment were obtained by sitting drop vapour diffusion using a protein concentration of 10 mg/ml and a precipitant solution of 100 mM sodium acetate pH 5.0, 1 M ammonium formate, 8% (w/v) poly-γ-glutamic acid (PGA-LM, Hampton Research). Larger crystals were obtained in 2 µl hanging drops and a precipitant solution of 100 mM sodium acetate pH 5.0, 0.7 M ammonium formate, 8% PGA-LM. Crystals of the laminin γ1 LN-LEa1–2 fragment were obtained by vapour diffusion in 200 nl sitting drops using a protein concentration of 9 mg/ml and a precipitant solution of 100 mM MES pH 6.5, 200 mM calcium acetate, 10% (w/v) PEG8000. Crystals were harvested in precipitant solution supplemented with 20% glycerol and flash-frozen in liquid nitrogen.

### Diffraction Data Collection and Processing

Diffraction data were collected at 100 K at beamline IO4 of the Diamond Light Source (Oxfordshire, UK). The data for the laminin β1 LN-LEa1–4 structure were collected from a single crystal at a wavelength of 0.9173 Å using a PILATUS detector, integrated with MOSFLM (www.mrc-lmb.cam.ac.uk/harry/mosflm) and scaled with programs of the CCP4 suite [37]. The data for the laminin γ1 LN-LEa1–2 structure were collected from a single crystal at a wavelength of 0.9763 Å using a CCD detector (ADSC) and processed with XDS [38].

### Structure determination

The laminin β1 LN-LEa1–4 and γ1 LN-LEa1–2 structures were solved by molecular replacement with PHASER [39] using search models constructed from the laminin α5 LN-LE1–2 structure [17]. Sequence alignments were used to identify the conserved regions and the laminin α5 LN-LE1–2 structure was trimmed accordingly. The correctly positioned search models were rebuilt and extended with O [40] and refined with CNS [41], making frequent use of simulated annealing omit maps to minimise model bias. Free R-factor cross-validation was used throughout to monitor the progress of refinement. Judging by the free R-factor, tightly restrained individual B-factors represented the best model of the disorder in our crystals. TLS refinement in PHENIX [42] did not substantially improve the free R-factor and was therefore not used. Inspection of the γ1 LN-LEa1–2 diffraction images showed that the effective resolution along c* was limited to ∼3.8 Å. The data set used for refinement was therefore subjected to ellipsoidal truncation and anisotropic scaling using the Diffraction Anisotropy Server (http://services.mbi.ucla.edu/anisoscale) [43], resulting in an improved electron density map for model building. Refinement against the modified data set converged at a free R-factor of 0.275. A final round of refinement was carried out against the original, non-truncated, data set to 3.2 Å resolution, resulting in a final free R-factor of 0.297. The final laminin β1 LN-LEa1–4 model spans residues 29–492, with residues 113–117 and 284–289 missing; the C-terminal 50 residues are also disordered, although there is some density for the last disulphide-bonded loop of LEa4. The final laminin γ1 LN-LEa1-2 model spans residues 37–395, with residues 82–85 missing. At least one sugar moiety is visible at every predicted *N*-linked glycosylation site, and partial glycans were modelled in both structures. The laminin γ1 LN-LEa1–2 structure also contains a calcium ion. Crystallographic statistics are summarised in Table 2. The figures were made with PyMOL (www.pymol.org). The coordinates of the laminin β1 LN-LEa1–4 and γ1 LN-LEa1–2 structures have been deposited in the Protein Data bank (entry codes 4aqs and 4aqt, respectively).
